# Real-time Remote Expert-guided Echocardiography by Medical Students

**DOI:** 10.1186/s13089-023-00328-3

**Published:** 2023-06-02

**Authors:** Håvard Solvin, Matthias Lippert, Henrik Holmstrøm, Ole Jakob Elle, Henrik Brun

**Affiliations:** 1 Institute of Clinical Medicine, University of Oslo, Kirkeveien 166, 0450 Oslo, Oslo Norway; 2 The Intervention Centre, Technology and Innovation Clinic, Oslo University Hospital, Sognsvannsveien 20, 0372 Oslo , Oslo Norway; 3 Department of Informatics, University of Oslo, Gaustadalleen 23 b, 0373 Oslo, Oslo Norway; 4 Department of Paediatric Cardiology, Oslo University Hospital, Sognsvannsveien 20, 0372 Oslo, Oslo Norway; 5 Department of Paediatrics, Oslo University Hospital, Sognsvannsveien 20, 0372 Oslo, Oslo Norway

**Keywords:** Telemedicine, Remote, Remote-guided echocardiography

## Abstract

**Background:**

Echocardiography is a highly specialised examination performed by experienced healthcare professionals. These experienced healthcare professionals may not be available to patients during all hours in rural healthcare facilities. Remote-guided echocardiography could improve the availability of specialised care for patients living in rural areas. This study examined the feasibility of real-time remote guidance for medical students to perform an echocardiographic assessment of the left side of the heart. Thirteen healthy volunteers were recruited for remote-guided echocardiography, which was performed by 13 medical students. Student examinations/images were compared to reference echocardiography. Measurements of left ventricular fractional shortening and mitral valve blood flow velocity were also compared. Furthermore, guidance through a smartphone videoconference was compared to designated remote guidance software.

**Results:**

Two-thirds of the images acquired by students were rated as medium or good quality and usable to evaluate two thirds of the cardiac structures. No significant bias was found for left ventricular fractional shortening. The measurements from the students’ exams had a variation coefficient of 14.8% compared to the reference. The calculated deviation of the insonation angle was above 25° for both E and A-wave mitral valve blood flow velocity measurements. Images acquired by guidance through smartphone videoconference were of lower quality than those obtained using the designated remote guidance software.

**Conclusion:**

Real-time remote-guided echocardiography performed by medical students has limited value for clinical screening but could be useful for educational purposes.

**Graphical Abstract:**

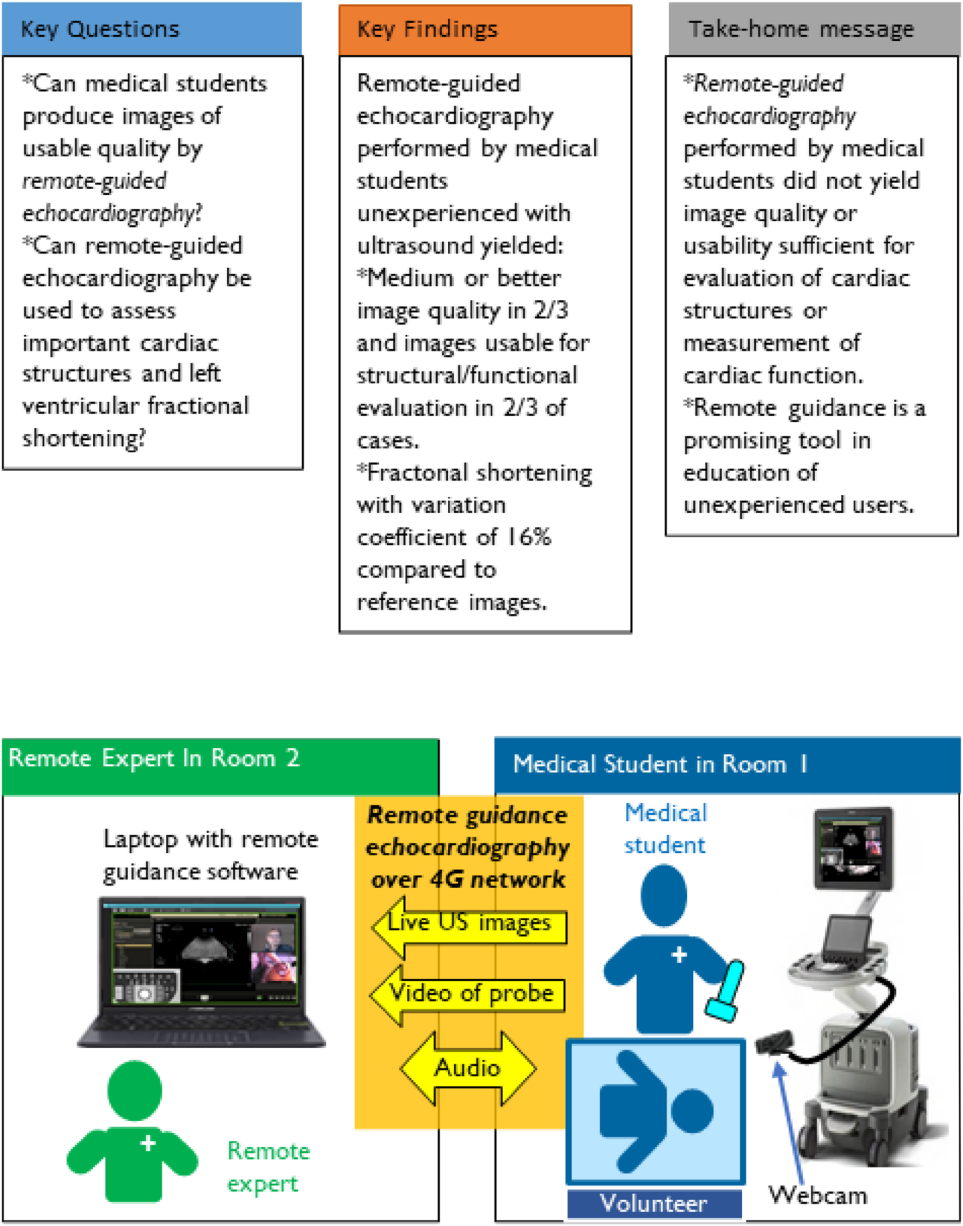

## Introduction

Echocardiography is a highly specialised examination performed by experienced healthcare professionals. Echocardiographic examinations performed by examiners without proper experience may yield false-positive and false-negative results, with significant implications for patient treatment and prognosis. The Canadian Society of Echocardiography requires echocardiographers to perform a minimum of 150 echocardiograms before independent work can commence [1]. Despite this, the evaluation of fellows shows that the diagnostic sensitivity for certain diagnoses is low [2]. Doctors on call in smaller hospitals frequently encounter situations where the necessary echocardiographic expertise is lacking. Remote real-time remote-guided echocardiography is a possible solution. An expert in a central hospital or at home can remotely guide the echocardiography performed at a local hospital. The platform frequently in use to perform remote guidance echocardiography today is smartphone videoconference, which may be suboptimal.

Different hardware setups for remote guidance ultrasound were evaluated by Smith et al. in 2018 who concluded that using a fixed camera, live transfer of ultrasound images, and verbal communication was superior to smartphone guidance [3]. Levine et al. evaluated transferral of ultrasound recordings made by remotely guided*,* non-physician healthcare workers. Images transferred by Facetime to an Apple iPad were rated as non-inferior compared to the ultrasound machine’s original images [4]. Several clinical studies on remote guidance cardiac ultrasound have been performed with different cardiac measurements/imaging and across different levels of prior cardiac ultrasound experience [5]. In 2017, Kim presented the results of remote guidance of 60 *echo-naive participants* performing a qualitative assessment of left ventricular contraction compared to expert Simpson biplane measurement of ejection fraction. Almost half of the participants could not obtain an apical four-chamber image of acceptable quality to visually estimate ejection fraction, and 13.3% could not produce an *apical four-chamber image* [6]. In 2014 Russel studied acquisition of a parasternal long-axis view by novice medical students through remote guidance using Google Glass. The control group receiving no guidance acquired significantly worse image quality and the images were not adequate for measurement of E-point septal separation (EPSS), while the remote-guided group and expert examination showed similar imaging results [7]. A more extensive remote interpretation study protocol was done by Jacobsen in 2022, to evaluate the feasibility of prehospital use of paramedic-performed echocardiography for risk stratification of patients with chest pain. Five different cardiac views were acquired and evaluated with good image quality score [8]. Bansal evaluated remote guidance in another educational setting. There were only minor differences in image quality when comparing echocardiograms performed after six hours of echocardiographic training through remote guidance with those performed after on-site training. No differences were found in the diagnostic sensitivity and specificity. Six hundred and sixty echocardiograms were evaluated [9].

To the best of our knowledge, there have been no previously published reports on real-time, remotely guided, complete echocardiography of the left heart performed by novice medical students. This study aimed to evaluate the feasibility of remote guidance in performing a screening-type echocardiographic examination of the left heart. The primary objective was to compare the echocardiography performed by remote-guided medical students to reference examinations through analysis of image quality, usability and examination time. The secondary objective was to compare image quality and examination time from student examinations guided by smartphone to examinations guided by designated software.

## Material and methods

Fourteen medical students were recruited after formal invitation posted on a website and email correspondence to perform remotely guided echocardiography. Any other selection was not done apart from the exclusion criterion of no additional ultrasound experience other than what is taught in their education. One student was excluded due to COVID-19 restrictions. All the students had undergone a cardiac anatomy course as part of their education. Out of 13 participants, seven had completed a brief practical course on ultrasound, including echocardiography, as part of their education. Thirteen healthy volunteers were recruited from acquaintances and a group of students. Of the 13 participating students, 11 were healthy volunteers after performing remote-guided study echocardiography.

**Fig. 1:**
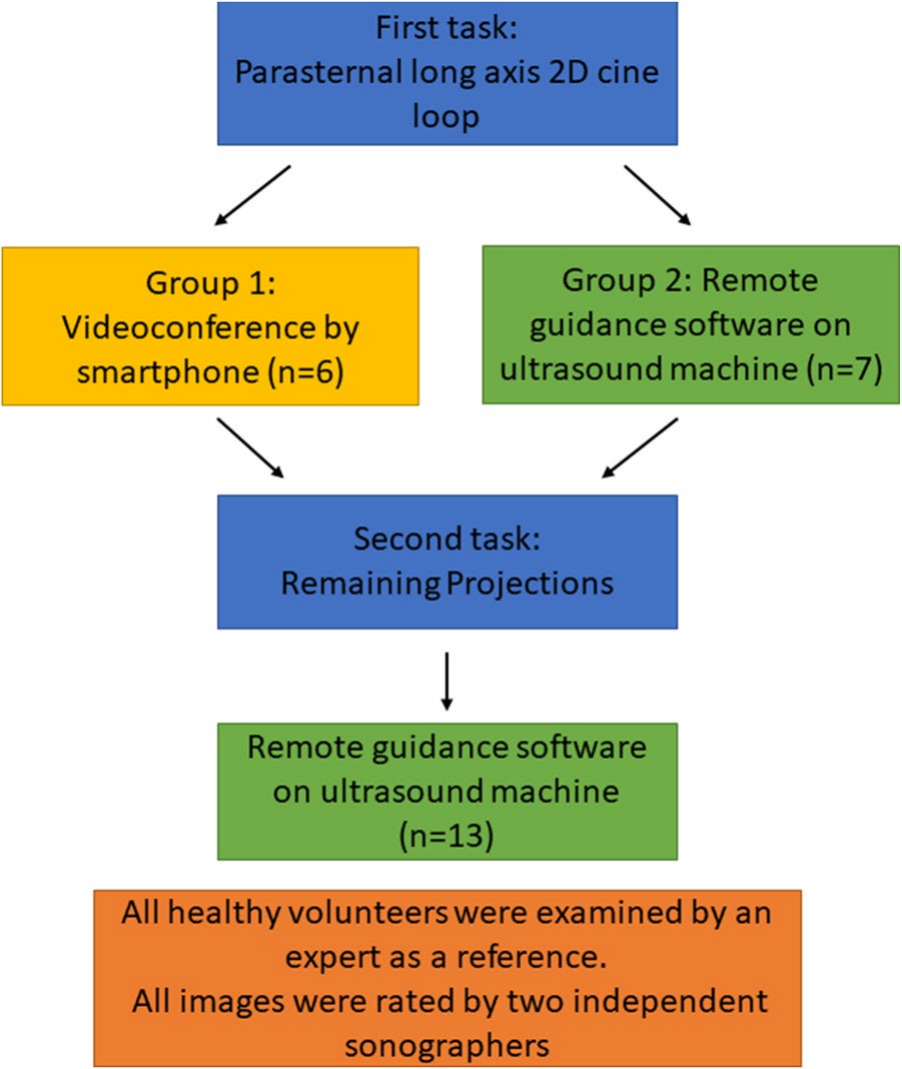
Graphical representation of study task flow

The students were randomly assigned into two groups for the first task of obtaining a parasternal long-axis 2D cine loop. Group one received remote guidance through a smartphone videoconference, and group two received guidance through designated remote guidance software on the ultrasound machine. In the second task, all students received guidance using remote guidance software to obtain five specific ultrasound images from the study protocol. These five images were: (1) parasternal long-axis colour Doppler cine loop of the mitral and aortic valves, (2) parasternal short-axis 2D cine loop of the left ventricle, (3) apical four-chamber 2D cine loop, (4) apical four-chamber colour Doppler cine loop of the Mitral valve, and (5) pulsed wave Doppler of mitral flow including measurements of E- and A-wave velocities. See Fig. 1.

The instructor (HS) provided an introductory lecture of 30 min on echocardiography and the basic settings of the ultrasound machine. Directly after, the students started performing echocardiography according to the protocol.

The students were remotely guided by the first author (HS), who is a resident doctor of paediatrics with 1 year of echocardiography training. The guide was positioned in a separate room, without direct contact with the students. All voice communication was done by standard mobile phones. Echocardiographic images were streamed in real-time to the laptop of the guide via a 4G network. Based on the guide’s continuous assessment of the ultrasound stream, real-time instructions were provided to the students on the movement of the probe according to predefined movement terminology. The aim was to acquire the correct images and complete the study protocol within an acceptable time frame. For the smartphone group in task 1, a Zoom (Zoom Video Communications, Inc., San Jose, CA, USA) videoconference was set up, with a 5.7-inch screen phone mounted on a separate stand, showing the screen of the ultrasound machine. The guide could not see the probe position during the exam for the smartphone group. Furthermore, the remote guidance group received guidance through REACTS software (Philips, Amsterdam, Holland), a designated software for remote guidance. The software streams the ultrasound image and a separate webcam showing the probe position for probe position guidance purposes to the laptop of the guide. This is illustrated in more detail in the Graphical Abstract. All healthy volunteers were placed in the left lateral decubitus position to optimise the acoustic windows.

The time spent for each acquisition was measured from when the probe touched the volunteer’s skin to when the store button was pressed. The image loops were recorded using retrospective capture for the students and prospective capture for reference echocardiography. No upper time limit was set. The images were stored as raw DICOM data on the ultrasound machine and transferred to the echocardiographic server of the hospital, where they were accessed for quality evaluation. The diagnostic image quality for each projection was rated by two independent and experienced sonographers on a scale of 0–3. A rating of 0 was «not usable quality», 1 was «bad quality», 2 was «medium quality», and 3 was «good quality». For each image, the two sonographers also answered a yes/no questionnaire regarding the usability of the assessment of the selected cardiac structures.

Reference examinations were performed by an experienced echocardiographer (ML) and served as the gold standard for all measurements. All measurements of left ventricular fractional shortening were derived from the 2D parasternal short-axis images and measured by ML. The measurements were performed 3 months after the examinations using EchoPAC software v.204 (General Electric Company, City of Boston, USA). All image reviewers were blinded to the examination type. End-diastole was defined by the R-wave of the QRS complex, and the systolic measurement was performed on the image with the smallest diameter of the left ventricle. All measurements were performed in triplicate, and the mean value was calculated. Finally, the fractional shortening measurements were reviewed for correctness by a second expert (HB), blinded to patient ID and examination type. In a few cases, the measurements were corrected before statistical analysis.

### Equipment

The ultrasound machine used was a Philips EPIQ 7G with cardiac software, software release 7.0.5 (Koninklijke Philips, Amsterdam, Netherlands). The image reading software used was ComPACS (MediMatic Srl, Genova, Italy) and the EchoPAC software plugin v.203 (General Electric Company, City of Boston, USA). The webcam used with the Phillips EPIQ to show probe position during remote guidance was a Logitech C920S PRO HD WEBCAM, Max Resolution:1080p/30 fps–720p/30 fps, Camera megapixel: 3 (Logitech, Lausanne, Switzerland). The smartphone used for remote guidance was a Sony Xperia L3 with a dual camera 13 MP, f/2.2, 26 mm (wide), 1/3.0″, PDAF 2 MP, f/2.4, (depth) (Sony Corporation, Tokyo, Japan). The remote guidance software used on the Phillips EPIQ was Reacts and Collaboration Live, INNOVATIVE IMAGING TECHNOLOGIES INC., and Reacts (Montreal, Quebec, Canada) (Koninklijke Philips, Amsterdam, Holland). The smartphone was connected to the laptop of the guide via Zoom. Both the smartphone and ultrasound machine guidance software were connected to a ASUS ROG STRIX G laptop (ASUSTeK Computer Incorporated, Taipei City, Taiwan) (R.O.C.). Data were analysed using STATA 16.0 (StataCorp LLC, Texas, USA). The network router used was the Huawei H138-380 wireless 4G router **(**Shenzhen, Guangdong Province, China).

### Student experience

The student experience was rated with a questionnaire with answers on a 6-point Likert scale. The questionnaire covered their subjective ability to solve the task, their evaluation of the communication with the guide, their ability to orient in the ultrasound image, their ability to get to the correct image, and their level of stress or relaxation during the remote guidance examination.

### Statistics

Student demographics were analysed using a two-sample Student’s *t*-test with bootstrap to compare the number of semesters per student group. The proportion of students with the ultrasound course as part of their education was analysed by a proportion test.

To evaluate the agreement and variation in left ventricular ejection fraction between student and reference-acquired images, we used a two-sample Student’s *t*-test, Bland–Altman plot, and variation coefficient. The image quality was compared using a two-sample Student’s *t*-test. The agreement of reference versus student visualisation of structures was analysed using Cohen’s Kappa.

The level of two-sided significance was 0.05.

## Results

### Student demographics

In the first task, there were no statistically significant differences between the two groups of students concerning ultrasound training (50% versus 57%, *p* = 0.8) and the present study semester (6.3 vs. 5.6, *p* = 0.6). Separating the students by ultrasound training showed a small difference in mean image quality (1.5 vs. 1.7, *p* = 0.4) and total assessable cardiac structures (9.5 vs. 10.1, *p* = 0.8) in favour of the student group who had the ultrasound training course, although insignificant. The group with the ultrasound training course used an insignificantly greater time on average (1043 s vs. 995 s, *p* = 0.8). The male/female ratio was 3/10, which was close to the gender representation of Norwegian medical students.

**Fig. 2:**
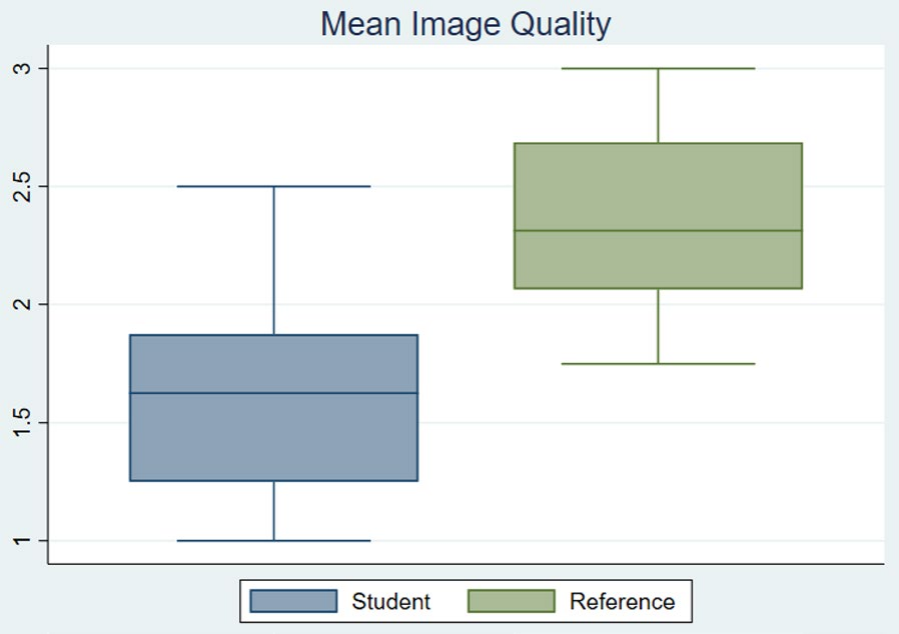
Mean image quality of students and reference on a scale where “0” is not usable, “1” is low, “2” is medium, and “3” is high image quality

### Sonographers’ assessment of remote-guided echocardiography versus reference echocardiography

The reference examinations had significantly better image quality compared to the student examinations, with a score of 2.37 versus 1.62 (*p* < 0.005) (Fig. 2). The mean examination time for students was 17 min and 6.1 times longer than that of the reference (*p* = 0.0000).

**Fig. 3:**
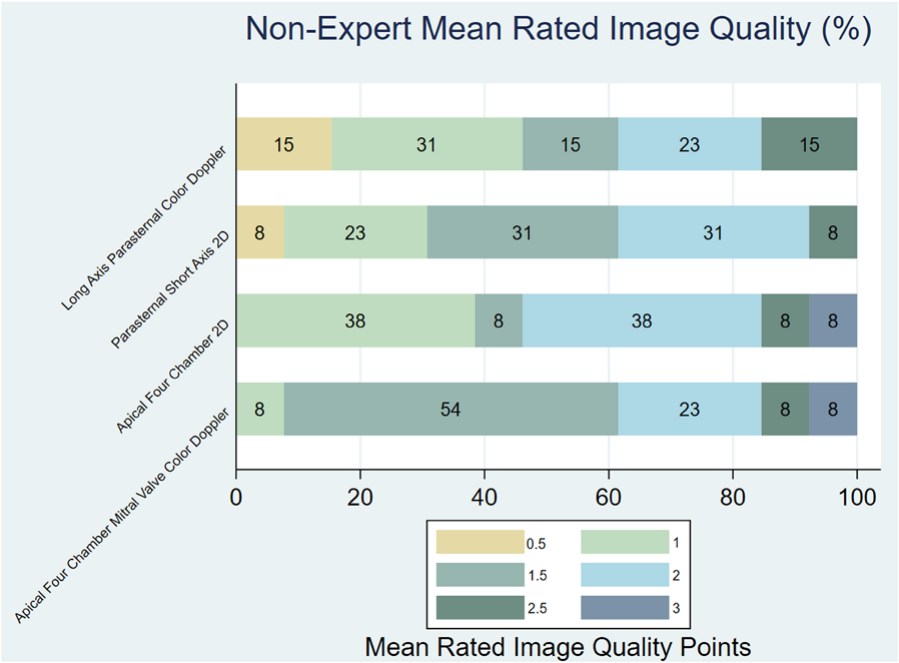
The mean rated image quality through for images two to four in the study protocol. Where “0” is not usable, “1” is low, “2” is medium, and “3” is high image quality

There seems to be a trend of better image quality towards the end of the study protocols, though not statistically significant when analysed by *t*-tests.

Both sonographers judged approximately one-third of the student images as not usable or bad quality. Data is shown in Fig. 3.

**Fig. 4:**
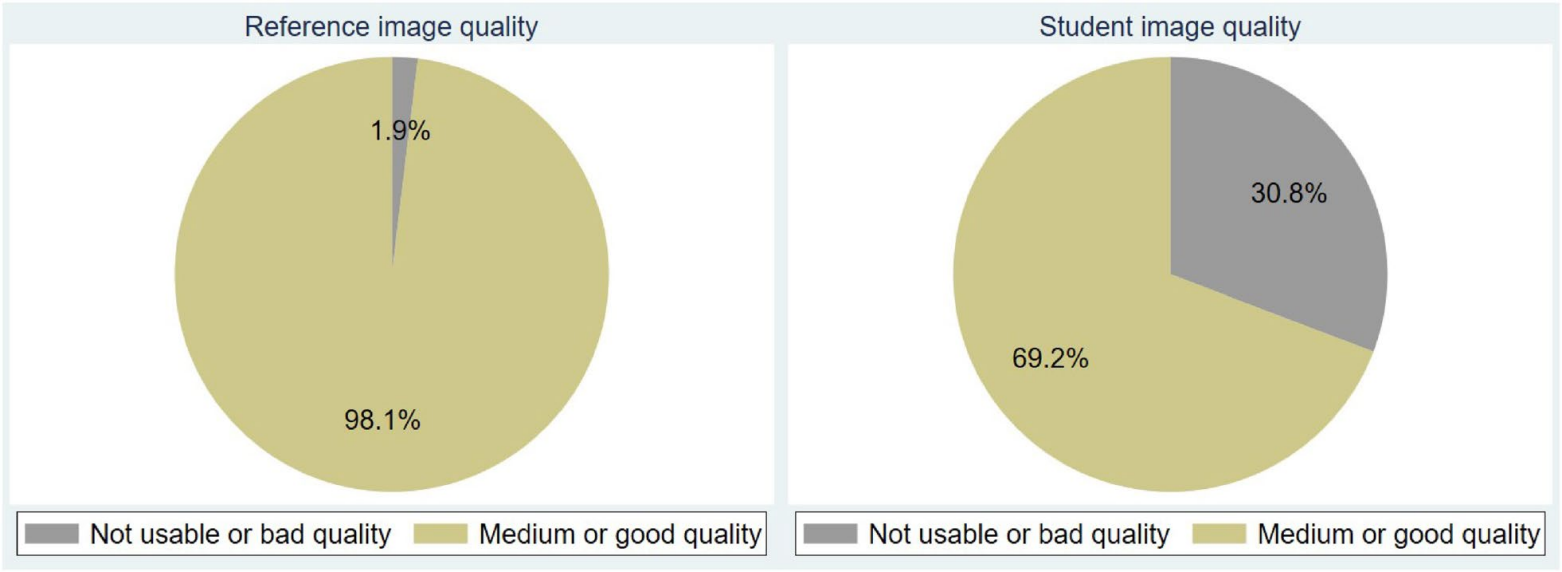
Diagrams showing the proportion of images with medium or good images for both reference and student examinations

**Fig. 5:**
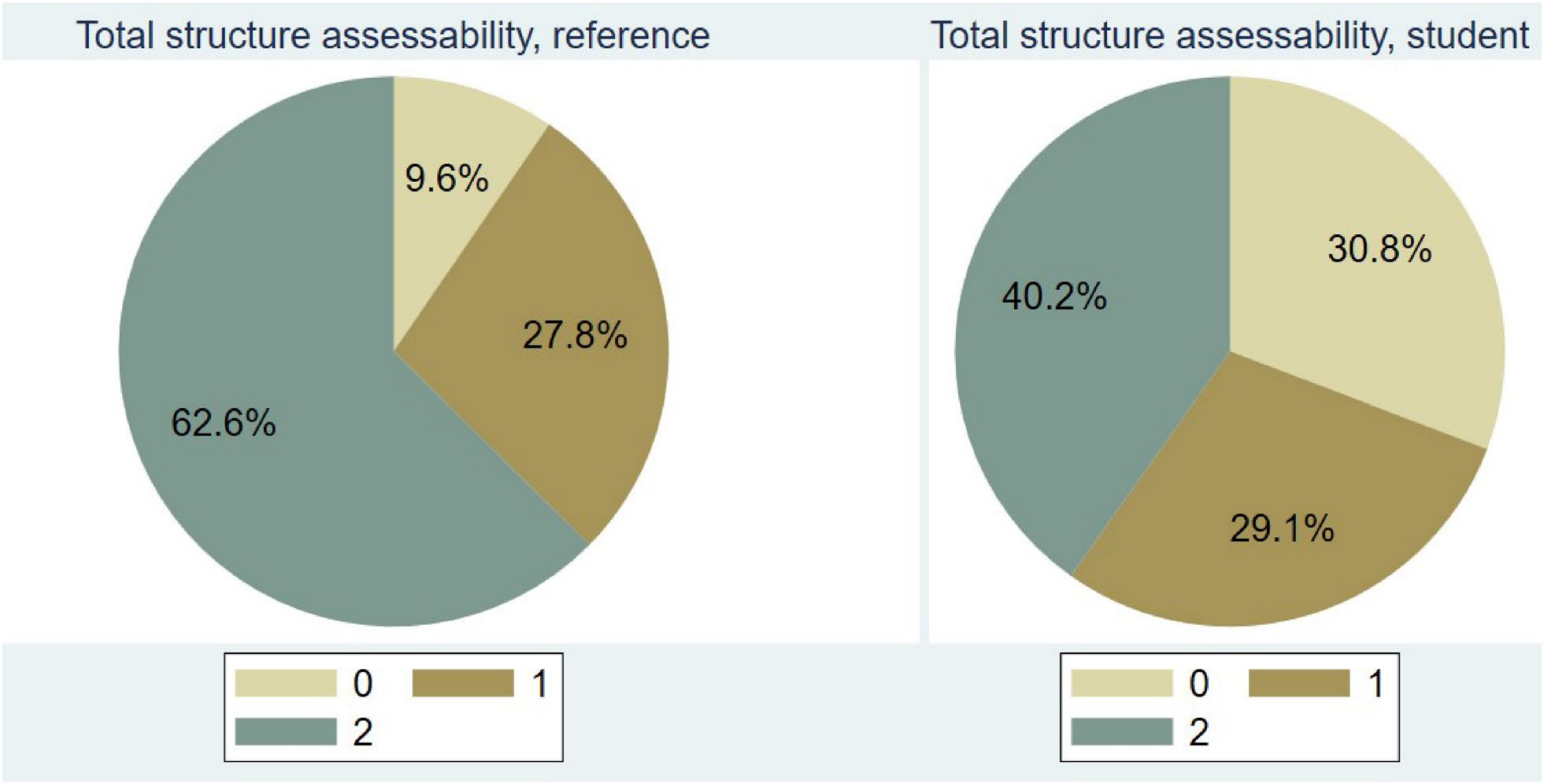
Diagram showing total structure usability for assessment by sonographers’ evaluation, in task two. 0—not assessable, 1—rated assessable by one sonographer, and 2—Rated assessable by both sonographers

Approximately one-third of the selected cardiac structural and functional assessments were judged as unusable for assessment by both sonographers when evaluating the student images. See Fig. 4. Approximately 40% were assessed as usable for assessment by both sonographers. See Fig. 5.

The student’s ability to image structures compared with reference imaging is shown in detail in Table 1 using Cohen’s Kappa analysis. Values show large variation, from negative values, suggesting worse agreement than random variation and towards 0.5, suggesting moderate agreement. The best agreement was reached for colour Doppler assessment of the aortic valve in the parasternal long-axis view and evaluation of left ventricular dimension and longitudinal contraction in the apical four-chamber view.

**Table 1:** (Qualitative as﻿sessments)—Agreement between qualitative assessment of remote-guided echocardiography versus reference

Structure function/dimension	Cohens Kappa
Colour Doppler parasternal long-axis	
Aortic valve	0.54 *p* = 0.024
Mitral valve	0.15 *p* = 0.253
2D parasternal short-axis, papillary muscle level	
The correct level of plane	− 0.35 *p* = 0.918
Correct angle of plane (circular ventricle)	− 0.02 *p* = 0.540
Right ventricular dimension	− 0.22 *p* = 0.800
Left ventricle segmental contraction	0.09 *p* = 0.358
2D apical four-chamber	
Left ventricular dimension	0.35 *p* = 0.048
Left ventricular longitudinal contraction	0.45 *p* = 0.026
Colour Doppler apical four-chamber	
Mitral valve	− 0.15 *p* = 0.795

### Left ventricular fractional shortening (FS)

The standard deviation between student FS and reference FS was 4.7 (range of deviation − 8 to 10) with a corresponding variation coefficient of 14.8%. See Table 2.

**Table 2:** Fractional shortening measured from reference and student examinations and their differences, with standard deviations

	Mean (± CI)	Standard deviation
Student FS (%)	31.6 (28.6–34.6)	4.9
Reference FS (%)	31.4 (28.3–34.5)	5.1
Difference	0.24 (*p* = 0.85)	4.7

**Fig. 6:**
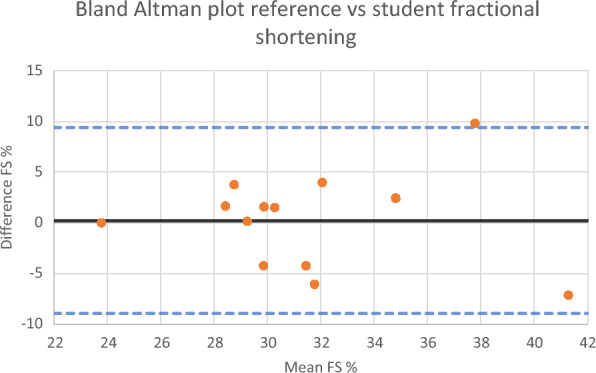
Bland–Altman plot comparing reference versus student-derived fractional shortening of the left ventricle in parasternal short-axis

The Bland–Altman plot shows that there was no systematic error in the variation between the fractional shortening from the student and reference examinations. See Fig. 6.

### Pulsed Doppler measurements

As part of the study protocol, the mitral valve peak E and A-wave velocities were also measured. A statistically significant difference between the student and reference groups was only found for the E-wave velocity. The following equation was used to estimate the angle deviation of Doppler insonation for the peak E- and A-wave velocities:

$$\cos x = V_{{{\text{Student}}}} /V_{{\text{Expert }}} \to x = \cos^{ - 1} \left( {V_{{{\text{Student}}}} /V_{{{\text{Expert}}}} } \right)$$

Based on the difference in the peak A- and E-wave velocities, the corresponding mean angle differences between the reference and student images were 26 and 28°, respectively. Data is shown in Table 3.

**Table 3:** Angle deviation of student versus reference Doppler velocity interrogation

Pulsed Doppler measurement	Student mean (mm/s)	Reference mean (mm/s)	Student/reference ratio
Mitral valve E-wave velocity	82.5 (76.1–90.0)	93.6 (81.3–105.9)	0.90 (0.82–0.99) *p* = 0.0274
Mitral valve A-wave velocity	50.2 (42.9–57.4)	55.7 (47.7–63.7)	0.94 (0.78–1.10) *p* = 0.4052

### Use of smartphone versus remote guidance software (RGS)

The reference examinations achieved significantly better image quality and spent less time than both guidance groups. The smartphone group had a significantly lower student-reference-time ratio than the RGS group. In contrast, the image quality in the RGS group was significantly higher. The mean time for the smartphone group was 162 s, compared to 374 s in the RGS group. The data are listed in Table 4.

**Table 4:** Use of smartphone versus remote guidance software (RGS)

Parameter	Phone group *n* = 6	RGS group *n* = 7	Difference
Student-reference time ratio	3.1 (0.99–5.12)	5.7 (3.7–7.8)	2.65 (0.06–5.23) *p* = 0.05
Student-reference image quality ratio	0.53 (0.35–0.72)	0.73 (0.66–0.79)	0.19 (0.03–0.35) *p* = 0.02

The student results were weighted against the reference performance for the same healthy volunteer. No significant differences or trends were found between the groups by a proportions test in the ability to assess the sizes of the left atrium and ventricle and show the anatomy of the aortic and mitral valves from the images.

**Fig. 7:**
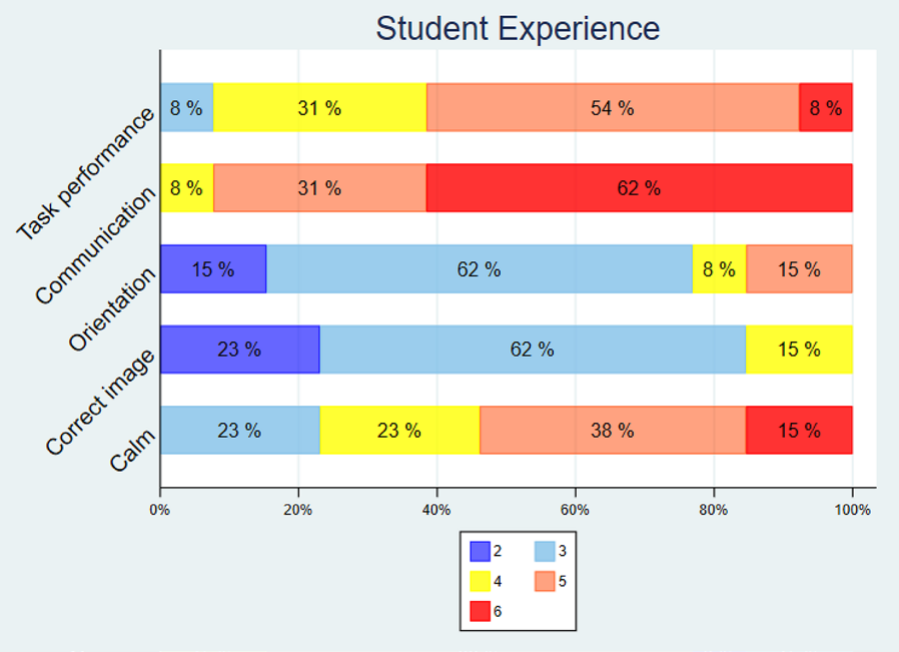
Student answers to a 6-point Likert scale. Task performance: 1—terrible, 6—great, Communication: 1—terrible, 6—great, Orientation: 1—nearly impossible, 6—unproblematic, Ability to get to correct image: 1—nearly impossible, 6—unproblematic, Feeling stressed or calm: 1—very stressed, 6—very calm

### Student experience

Only 8% of students rated their task performance as 3/6, and 92% rated their task performance as 4/6 or above. All students rated the communication with the guide 4/6 or better. Only 23% rated their ability to orient in the ultrasound image as 4/6 or better. A total of 85% rated their ability to get to the correct ultrasound image as 3/6 or lower. All students rated their feeling of being calm as 3/6 or better (Fig. 7).

## Discussion

This study aimed to assess the feasibility of remote guidance for medical students to perform an echocardiographic assessment of the left heart by assessing the examination time, image quality, and ability to assess cardiac structures.

Main findings:

- Remote-guided medical students were able to produce images of medium or good quality that were usable for the assessment of cardiac structures through remote guidance in approximately two thirds of the images and structures.
- The variation coefficient for remote-guided left ventricular fractional shortening was 14.8%.

If we consider the remote guidance of medical students as a diagnostic test for functional or structural anomalies, the maximum sensitivity that we can theoretically achieve based on the results of this study is two thirds. However, this level of sensitivity would not be sufficient for use in a clinical setting. It was anticipated that there would be a difference in the quantitative analysis performed by the sonographers for image quality and the ability to assess cardiac structures between the students and the reference examinations. The highest level of agreement between the reference and the student’s ability to image selected cardiac structures was found for colour Doppler evaluation of the aortic valve in the parasternal long-axis view and when evaluating the left ventricle in the apical four-chamber view*.* The high score for the Doppler evaluation of the aortic valve might be due to a learning effect from previous imaging of the same projection in 2D, indicating that the method may be of interest from a teaching perspective. The relatively good results for the usability of the apical four-chamber projection in assessing the size and longitudinal contractility of the left ventricle could be due to the simplicity of the apical four-chamber image, which may be the main association with echocardiography for many students and doctors. The results of imaging structures and image quality in the parasternal projection showed a trend to be poorer compared to results from the apical projection, although not statistically significant. From our experience it is hard for novices to place the probe close enough to the sternum to yield good-quality parasternal images. Even for the reference examinations, approximately 10% of cardiac structures were scored as not assessable, which might be due to poor patient-specific image quality and high focus to complete a good-quality examination in the shortest time possible.

In the quantitative assessment, the comparison of fractional shortening showed statistically insignificant bias by Bland–Altman analysis and paired Student’s *t*-test, with a considerable variation coefficient. Considerable deviation of insonation angle between student and reference acquisition was found for both the peak E and A-wave velocities. Compared with the precision of earlier publications, the student measurements do not quite hold up to clinical standards with a variation coefficient of approximately 5–6% and 5% for M-mode left ventricular ejection fraction and peak velocity in mitral stenosis, respectively [10, 11]. This echocardiographic protocol was designed for a screening of left heart anatomy and function based on recent guidelines from the American Society of Echocardiography [12]. Considering both quantitative and qualitative assessments, the results suggest that the use of remote-guided students without basic echocardiographic training is not yet appropriate to perform a diagnostic evaluation. Evaluation of novices with basic echocardiographic training or narrowing of the protocol to focus on one projection or functional measurement could have yielded higher image quality and usability, as shown in earlier studies [7]. The results from this study of remote guidance show that students can visualise cardiac structures, even though it is their first or second time performing basic echocardiography. In addition, there seems to be a non-significant trend of better imaging results throughout the study protocol which may suggest that remote guidance is a feasible tool for echocardiographic training.

Considering smartphone guidance, the results might be due to the guide’s tendency to accept lower-quality images. The fact that the examination time was considerably shorter could be due to the difficulty of using videoconference with poor quality of video feed to guide the student. This might introduce a bias in the image quality for this image acquisition task in favour of the remote guidance software. An alternative study design can apply a fixed examination time for both methods.

The students’ feedback on the questionnaires showed that they were not stressed during the examination and felt that communication with the guide worked well. They were somewhat more self-critical on the questions regarding their ability to produce the correct images, orient in the ultrasound image, and their overall ability to solve the task. One way to interpret these results is that the students found the task quite challenging, but that remote guidance yields an acceptable user experience for initial echocardiographic training.

The sonographers assessed the usability and quality of the examinations by evaluating two or three heart cycles of saved loops. There was a substantial amount of time spent scanning between the stored images, during which the students attempted to obtain the correct images. A lot of information was not shown to the evaluating sonographers in this study; however, information was shown to the guide of the examination and would have been used in a clinical assessment. The evaluation of a continuous recording of the examination could have resulted in better image quality and usability for the assessment of cardiac structures. Naturally, the reference echocardiographer was better at showcasing function and morphology in the stored loops than the students, being new to echocardiography and operating an ultrasound machine.

### Study limitations

All healthy volunteers were below 30 years of age, thus reducing the generalisability of the results to patients with different characteristics. The models used in this study does likely have better patient-specific image conditions compared to the average patient in need of echocardiography, leading to a bias. Furthermore, the students volunteered and thus might be more interested in echocardiography compared to the average medical student and could introduce a bias in favour of remote guidance in the results of this study. Evaluating usable image quality is an indirect measure that is not directly applicable to the diagnosis of pathology. The questionnaire for qualitative assessment has not been previously validated. The evaluators who completed the questionnaires were sonographers and might have been more stringent with images that did not look like the schoolbook example. Physicians might be more concerned with imaging structures and functions in a clinical setting. Theoretically, physician evaluation of images might have provided slightly better results. Due to the small sample size (*n* = 13), caution must be exercised when drawing conclusions from the data. There was a discrepancy in the echocardiographic experience between the guide of the students and the reference echocardiographer, introducing a possible bias. The image acquisition task is basic, so we hypothesised that the effect of the difference in echocardiographic experience would be negligible compared to the effect of echocardiography performed by remote-guided novices.

Previous studies have assessed the feasibility of remote guidance echocardiography by evaluating image quality and ventricular function by comparing “eyeballing” with conventional echocardiographic measurements [6]. This study is novel in that it evaluates a remote guidance platform to guide novice medical students and uses well-established and validated echocardiographic measurements to assess the agreement between reference and remote-guided echocardiography. In line with previous publications, this study demonstrates suboptimal performance for remote guidance of inexperienced users in clinical use [6, 13, 14]. However, our results could be poor due to the complexity of the study protocol and the low experience of the medical students, not due to the remote guidance itself. For educational uses, remote guidance may be a very useful tool, as shown by Bansal, and supported by a non-significant trend of better mean image quality towards the end of this remote guidance study protocol [9].

## Conclusions

Remote guidance of novice medical students performing an echocardiographic screening protocol for left heart function resulted in suboptimal image quality and structure assess ability. The designated guidance software showed better image quality than the smartphone guidance. The results could be considered hypothesis generating for further development of techniques for remote-guided echocardiography and its use in education, towards making specialised care more available to patients living in rural areas.

## Data Availability

The datasets used and/or analysed during the current study are available from the corresponding author upon reasonable request.

## Abbreviations

RGS: Remote guidance software
FS: Fractional shortening
